# Employee Stress, Reduced Productivity, and Interest in a Workplace Health Program: A Case Study from the Australian Mining Industry

**DOI:** 10.3390/ijerph16010094

**Published:** 2018-12-31

**Authors:** Tamara D. Street, Sarah J. Lacey, Klaire Somoray

**Affiliations:** Wesley Medical Research, Brisbane, QLD 4066, Australia; slacey@wesleyresearch.com.au (S.J.L.); KSomoray@wesleyresearch.com.au (K.S.)

**Keywords:** work stress, productivity, impairment cost, stress management, employee characteristics, workplace health promotion, health and safety

## Abstract

The Australian mining sector has an elevated industry prevalence of stress and high stress related productivity impairment costs. This study surveyed 897 employees from an Australian mining company to identify characteristics associated with: (a) high stress related productivity impairment costs; and (b) likelihood of stressed employees wanting stress management assistance at work. Groups associated with average annual productivity impairment costs in excess of $50,000 per employee included: permanent day shift employees; employees who reported being stressed at work most of the time; employees who reported being stress at work all of the time; and employees who were contemplating better managing their stress in the next 6 months. Overall, 52% of employees who identified as being in the contemplation stage of change for stress management and 52% of employees who experienced stress most of the time reported wanting stress assistance with stress. However, only 33% of stressed permanent day shift employees and 36% of employees who experienced stress all the time reported wanting stress assistance. To achieve a high return on investment when implementing workplace stress management programs in the mining industry, practitioners need to strategically target health promotion to engage stressed employees with high productivity impairment costs and low desire for stress management assistance.

## 1. Introduction

Stress management refers to the act of engaging in deliberate strategies to control ones level of stress, particularly chronic stress. Managing employee stress is a priority for advancing worker health in the global mining industry. An elevated industry prevalence of stress and the high associated personal and organisational costs of stress indicates a need for workplace health and safety risk management action. In Australia where this study was conducted, research has identified that psychological distress is significantly more prevalent in Australian mining workforces than in the general Australian population [1,2]. More specifically, in an adult Australia population sample, 11.7% of respondents had Kessler Psychological Distress Scale (K10) scores that indicated high/very high psychological distress [3]. Comparatively, 28% of employees from mine sites in South Australia and Western Australia [1], and 12.7% of employees from mine sites in New South Wales and Queensland [2] had K10 scores indicating high/very high psychological distress.

High economic costs of stress related absenteeism and presenteeism have been reported in the Australian mining sector. For example, in a Queensland mining company work-related stress accounted for the highest financial burden (compared across 25 medical conditions) [4]. Employees who reported experiencing stress at work were 19% less productive than employees who did not report experiencing stress [4]. Furthermore, based on productive time lost calculations from Queensland and New South Wales mines it has been estimated that psychological distress has an annual economic cost of $153.8 million to the Australian coal mining industry, representing almost 9% of pre-tax operating profit [5].

Work-related stress has been linked to detrimental employee health outcomes. For example, a longitudinal study spanning 14 years found that higher levels of workplace stress were associated with an increased risk of metabolic syndrome, a precursor of coronary heart disease [6]. Workplace stress has also been indirectly linked to employees’ health through the experience of stress being associated with greater engagement in negative health behaviours such as tobacco smoking, inadequate diet, insufficient physical activity, and alcohol use [7,8]. It has been speculated that conditions associated with mining employment including long work rosters and remote work locations may increase the risk of miners experiencing stress [9]. Concerns for miners’ health have been raised with several recent public enquires into work practices associated with the Australian mining industry, such as fly-in fly-out (FIFO) or drive-in drive-out (DIDO) arrangements, and impacts on mental health and suicide [10,11]. Some studies have found that Australian FIFO miners report work-life balance difficulties and relationship stress [12] and family stress associated with frequent miner absence from home [13]. McPhedran and De Leo [9] recommend caution when interpreting associations between mining employment and stress noting that work practices, rather than employment industry, had a direct relationship with some stress measures. In their comparison of male miners to males in other occupations, they identified that mine employees on average worked longer hours than employees in other occupations and working longer hours was independently associated with perceived lower quality family relationships and higher levels of relationship stress [9].

There are many workplace risk management strategies with research evidence of effectiveness [14,15]. Effective stress management has also been linked to improvements in other health behaviours. For instance, Lipschitz et al. [16] demonstrated that individuals who improved their stress management also increased their likelihood of exercising and managing their depression over a period of six months. A key challenge for health and safety practitioners is engaging stressed employees to proactively seek stress management assistance. This is particularly important given that low help seeking behaviours have been associated with the male-dominated mining industry [17].

Historically, studies have researched stress management strategies for adults assuming that anyone who requires assistance with stress management is ready to change their behaviour, with limited consideration to the actual readiness of the individual [18]. Although existing workplace based stress management studies have not specified the percentage of stressed employees who are prepared to adopt stress management practices, a population based study including 1085 adults recruited from national market research directories identified that at baseline measurement, over 80% of the sample were not ready to adopt stress management practices [18]. These individuals were not practicing effective stress management behaviours, including physical activity, regular relaxation, taking time for social activities, and/or talking with others, and not intending to start practing stress management strategies in the immediate future. Applying the Stages of Change Model of behaviour change, these adults were classified as being in a pre-contemplation or contemplation stage of change for stress management [18]. The randomised clinical trial study found that adults who participated in a Stages of Change Model stage-matched stress management intervention, as compared longitudinally to a control group, had significantly greater progress towards stress management action and maintenance behaviours, significantly lower stress levels, and were significantly more likely to be practicing healthy stress management and avoiding unhealthy stress management behaviours [18].

Within a mining workforce, research has identified that stage of readiness was not associated with likelihood of wanting assistance with reducing or quitting smoking [19], but was associated with wanting assistance with healthy weight management [20]. It was found that employees who were in the contemplative, preparation, and action stages for improving their eating habits were more likely to desire assistance for healthy weight programs compared to employees in the pre-contemplative stage. Employees in the action and maintenance stages for improving their physical activity habits were also more likely to desire assistance for healthy weight programs compared to employees in the pre-contemplative stage [20]. Research is needed within a mining workforce to identify the prevalence of employees ready to adopt stress management assistance and to explore whether stage of readiness is associated with stress impairment productivity costs and likelihood of seeking stress management assistance.

Although companies need to equitably provide health support for all employees, to maximize return on program investment targeted program promotion is critical to engage employees at risk of stress related health issues. In addition to understanding the employee characteristics associated with stress and readiness to change, practitioners would benefit from understanding if employee characteristics (including socio-demographic and work characteristic variables, e.g., age and job role) are associated with stress productivity costs and likelihood of seeking stress management assistance.

Research within the Australian mining sector suggests that certain employee demographic and work characteristics are associated with greater risk for psychological distress [1,2]. More specifically, Bowers et al. [1] found that mining employees aged 25 to 35 years and shift-work employees (rostered as two weeks on, one week off) were more likely to report psychological distress. However, it is unclear from the available literature which employee characteristics are associated with high stress-related productivity impairment costs. Similarly, empirical research indicates that females and middle-aged persons [21,22,23] are significantly more likely to access professional services to address stress management and other mental health concerns. Previous studies using a mining workforce also indicate that gender and age influence preference for health promotion programs [19]. For example, females and employees aged 24 years and under have been found to be more likely to want assistance for smoking cessation [19].

The limited available research to date suggests that employee characteristics and employee readiness for change may be associated with preference for health promotion programs such as those that target stress management. The aim of this study was two-fold: (a) To investigate, within employees’ who reported high levels of stress, the relationship between employee characteristics, stage of change for stress management, and productivity impairment costs; and (b) The relationship between employee characteristics, stage of change for stress management, and desire for assistance with stress management through a workplace health promotion program. Although this case study focuses on the Australian mining workforce, it is likely that the findings will have practical application for the development of stress management strategies in the global mining industry.

## 2. Materials and Methods

### 2.1. Participants

A sample of 897 employees from an Australian mining company were recruited to participate in the study. Participants were aged between 17 and 73 years. Consistent with the organizations workforce characteristics, the majority of participants (74%) were male. It was not possible to calculate a response rate due to the recruitment process, however the mining organisation confirmed that the sample was representative of the workforce demographic characteristics. Furthermore, variance in survey responses indicated that participants included a range of employees. Participant employment characteristics are detailed in Table 1. Reported percentages exclude missing data and are, therefore, calculated from different sample sizes as a few participants chose not to report some of their demographic and work information.

Of the 893 employees who responded to the stress item (*n* = 4 were missing data), 375 employees reported experiencing stress while at work ‘some of the time’ to ‘all of the time’ (refer Table 2). The majority of these stressed employees reported that they were not ready to adopt stress management practices, with 34.0% (*n* = 106) in the pre-contemplation stage and 22.8% (*n* = 71) in the contemplation stage. Of the stressed employees who were ready to adopt stress management behaviours, 6.4% (*n* = 20) were in the preparation stage, 27.2% (*n* = 85) in the action stage. The remaining 9.6% (*n* = 30) were in the maintenance stage, reporting that they were attempting to continue managing their stress. The sample of stressed employees was analysed to identify employee characteristics and stress management stages of change associated with (a) high productivity impairment costs and (b) desire for assistance with stress management.

### 2.2. Design

This study was approved by the by the Uniting *C*are Health Human Research Ethics Committee (#2013.03.74). The study utilized a cross-sectional design. Voluntary informed consent was obtained from each participant.

### 2.3. Procedure

The research team visited two Australian mining sites and a corresponding residential mine village. Due to safety regulations the team was prohibited from entering some work site areas. Company managers nominated working units with the goal of obtaining a representative sample of employees. In these work units, participant information sheets were displayed in common gathering areas and announcements were made by mangers at daily work group meetings. All employees of the selected work units who were sighted by the researchers during the data collection period were invited by the researchers to participate in the health survey. The survey was provided in hard copy (on paper) and once complete returned to the research team. All data were entered and analysed in IBM SPSS version 21 (IBM, Armonk, NY, USA). Participants included a mixture of operational, managerial, and administrative roles. Managers were not informed of which employees participated in the research.

### 2.4. Measures

Demographic and employment characteristic measures replicated government survey and corporate health survey items [4,24]. Consistent with previous research [4], stress was measured by the item “How much of the time in the past four weeks did you feel stressed while at work?” Response options included: none of the time; a little of the time; some of the time; a good bit of the time; most of the time; and all of the time. Participants were classified as stressed if they selected: some of the time; a good bit of the time; most of the time; or all of the time. Participants were classified as low risk of stress and excluded from stress analyses if they responded: none of the time; or a little of the time.

Consistent with previous research measuring miners’ readiness to change healthy behaviours using the Stages of Change Model [25], participants were asked to select one of five statements in response to the following question. ‘How would you describe your approach to stress management?’ Response option statements were amended from previously published nutrition and physical activity focused statements [25] to focus on stress management and were based on the traditional Stages of Change Model of behaviour change stage descriptions [26]. More specifically, stress management stage of change was measured by the selection of one of the following statements: pre-contemplative “As far as I’m concerned my stress management habits don’t need changing”; contemplative “I’m seriously intending to better manage my stress in the next 6 months”; preparation “I have definite plans to better manage my stress in the next month”; action “I am doing something to better manage my stress”; and maintenance “I took action more than six months ago to better manage my stress and I’m working hard to maintain this change”.

The Worker Productivity and Activity Impairment—General Health (WPAI:GH) questionnaire [27] items including hours worked, work absenteeism (i.e., work time missed due to health), and work presenteeism (i.e., impairment while working due to health) were replicated with minor amendments. Consistent with previous research using a shift work mining sample [4], the original seven-day measurement period was extended to a four-week period to minimize the impact of acute illnesses and shift work rosters to read, “During the past four weeks, how many days did you miss from work because of your health problems?”

Based on established World Health Organization and government survey items [24,28] the following outcome item “Would you like assistance with stress management?” was asked in the workplace health program survey section to measure employees’ preference for assistance.

### 2.5. Analyses

All analyses were conducted using the IBM software package SPSS version 21. Consistent with previous research [4], the current study followed the protocol outlined by Lenneman et al. [29]. However, it was not necessary to calculate ‘excess costs’ as the study was limited to a subset of employees who reported experiencing stress at work (*n* = 375). As presented by previous research [4], productivity impairment was calculated based on a modified version of the WPAI:GH. Specifically, productivity impairment was calculated for each employee who reported experiencing stress at work using the sum of days absent from work multiplied by 5 (which represents five days of work during a working week), and rate of presenteeism expressed as an overall impairment percentage. The following formula was used in calculating the work impairment percentage (1).

Formula:

Absenteeism impairment = days off work × 5 Presenteeism impairment = presenteeism score × (20 – days off work) Work impairment percentage = absenteeism impairment × presenteeism impairment(1)

Productivity costs were calculated by multiplying the overall work impairment percentage by the average Australian mining annual salary of $134,784 [30]. Finally, independent sample t-tests and a one-way ANOVAs were performed to examine the differences in productivity costs depending on employee characteristics and stage of change.

## 3. Results

### 3.1. Stress-Related Productivity Impairment Costs

Of the 375 employees who reported experiencing stress at work, 23.4% (*n* = 82) reported at least one day of absence from work due to personal health problems in the past four weeks. Twenty-five employees (6.7%) also stated a high degree of presenteeism, reporting that they had trouble concentrating at work or doing their best due to personal health problems ‘most of the time’ or ‘all of the time’ in the past four weeks. Overall, stressed employees were associated with an average of 33.6% work impairment and $45,240.70 (SD = 30,655.26) in productivity costs per employee.

Independent sample t-tests and a one-way ANOVA were performed to examine the differences in productivity costs depending on employee characteristics (see Table 2 and Table 3). Significant differences were identified between roster type, contract, and residency status. As seen in Table 2, employees on permanent day shifts were more likely to be associated with higher productivity costs compared to employees on rotating/alternating shifts, Cohen’s *D* = 0.41. Permanent employees were significantly more likely to report higher work impairment which was associated with higher productivity costs compared to contractors, Cohen’s *D* = 0.42. Employees who resided in the mining towns were also more likely to be associated with higher productivity costs compared to FIFO/DIDO employees, Cohen’s *D* = 0.35. Based on Cohen’s *D* conventions, these differences were considered small to medium. Productivity costs did not significantly differ between males and females and among the different age groups.

Productivity costs also significantly differed between self-reported levels of stress at work (see Table 3). A trend is observed in Table 3 with increased frequency of experiencing stress at work being associated with increased productivity costs. Employees who reported feeling stressed ‘all of the time’ in the previous four week period reported the highest productivity costs (*M* = $75,337.16; SD = $47,379.12). Post-hoc analysis using Games-Howell test were performed due to the unequal variances and sample sizes between the groups. Employees who reported experiencing stress ‘all of the time’ showed significantly higher costs compared to employees who reported only feeling stressed ‘some of the time’, *p* = 0.012 and the difference showed a large effect, Cohen’s *D* = 1.02. Employees who reported feeling stressed ‘most of the time’ were also associated with significantly higher productivity costs compared to those who only experienced stress ‘some of the time’, *p* < 0.001, Cohen’s *D* = 1.00 and ‘a good bit of the time’, *p* = 0.024, Cohen’s *D* = 0.53. The effect sizes were large and medium, respectively. Employees who reported feeling stressed ‘a good bit of the time’ were also associated with higher productivity costs compared to employees who only felt stressed ‘some of the time’ at work, *p* = 0.004, and the effect was small to medium size, Cohen’s *D* = 0.43.

Differences in productivity costs depending on stage of change for stress management were examined. Table 4 reveals that, on average, the highest productivity impairment costs were associated with employees classified in the contemplation stage of change for stress management while employees in the pre-contemplation stage were associated with the lowest average productivity impairment cost. A one-way ANOVA was conducted to assess the differences in productivity costs based on employees’ readiness to change their stress management behaviours. An overall significant difference was found, *F* (4, 307) = 6.78, *p* < 0.001. Post hoc analysis using Games-Howell test suggest that, contemplation employees were associated with significantly higher productivity costs compared to pre-contemplation employees, *p* < 0.001 and the difference had a medium to large effect, Cohen’s *D* = 0.77. Employees in the action stage were also associated with significantly higher productivity costs compared to pre-contemplation employees, *p* = 0.015. However, the effect size was only small to medium, Cohen’s *D* = 0.46. No other significant differences were found between the other groups.

### 3.2. Desire for Assistance with Stress Management

Of the 375 employees who reported experiencing stress at work, only 28% (*n* = 105) reported wanting assistance to manage their stress. A hierarchical logistic regression was performed to predict the likelihood for wanting assistance with stress management. Participants’ employee characteristics and reported levels of stress at work were included in Step 1 to partial out their effect in the regression model. The stage of change for stress management variable was included in Step 2. Step 1 was significant, χ^2^(8) = 39.76, *p* < 0.001, explaining 19.4% of the total variance (Nagelkerke *r*^2^ = 0.19) and correctly classified 76.4% of the cases. The model’s sensitivity was 37.7 and specificity was 91.4. As shown in Table 5, being female and having higher levels of stress were significant predictors of desiring stress management assistance. Female employees were twice as likely to want stress management assistance compared to male employees. Employees who reported experiencing stress more frequently were significantly more likely to report desiring stress management assistance compared to employees who reported experiencing stress less frequently. Age, shift work rotation, employment status, and current work arrangement were not significant predictors within the Step 1 of the model.

When the stages of readiness to change were added in the model, the second block was significant, χ^2^(4) = 32.65, *p* < 0.001, explaining an additional 13.9% of the total variance. Overall, the model was significant, χ^2^(12) = 72.42, *p* < 0.001 and explained 33.3% of the total variance (Nagelkerke *r*^2^ = 0.33) and accurately classified 78.2% of the cases. The model’s sensitivity was 49.4 and specificity was 89.4. Pre-contemplation employees were the reference category for the readiness to change one’s approach to stress management. As shown in Table 5, employees in all readiness stages were significantly more likely to desire stress management assistance than stressed employees who reported that their stress management habits do not need changing. However, it is important to note that the odds ratio’s lower confidence intervals for employees in the action stage nearly encompasses 1, indicating that being in the action stage may not necessarily increase the employees’ likelihood to desire assistance for their stress. The odds ratio of the action group in desiring assistance is also small (OR = 2.85) compared to employees in the pre-contemplation stage. Examination of the other groups revealed that employees in the contemplation stage showed the highest odds ratio, indicating that this group were 11.05 times more likely to want assistance for stress management compared to the pre-contemplation group. Furthermore, compared to the pre-contemplative employees, preparation employees were 7.13 times and maintenance employees were 5.22 times more likely to desire assistance for stress management.

## 4. Discussion

Within a mining workforce sample, this study identified employee characteristics and stress management stages of change associated with high stress-related productivity impairment costs and desire for assistance with stress management. Of the employees who reported experiencing stress at work, employee groups associated with significantly higher productivity impairment costs included: day shift workers; permanent contract employees; employees who reside within the mining towns; frequently stressed employees; employees intending to better manage their stress in the next six months; and employees who are actively managing their stress. Although previous research has reported that FIFO miners were at risk of stress due to their work arrangements [12,13] in the current study they had lower stress impairment costs than permanent day shift employees.

The lower productivity impairment costs associated with alternating rosters, contractors and FIFO/DIDO employment may be related to the roles and responsibilities associated with the different types of employees appointed to permanent day shifts versus alternating contracts. To protect participant anonymity the current study did not gather data regarding job position, however, the researchers are aware that within the current sample, employees working permanent day shifts included managers, professionals, administrative, and operational staff. Comparatively, employees with alternating rosters, FIFO/DIDO employment, and contractual work were more likely to be appointed to operational mining roles. A study by Ling et al. [5] showed that managers within the coal mining industry had higher average lost productivity time costs compared to machine operators and trade workers. Managers were also associated with higher psychological distress [2].

In this mining workforce sample, employees in the contemplation stage for stress management had the highest average annual stress-related productivity impairment cost ($53,761 per employee). Although individuals who are classified as contemplative intend to improve their behaviour in the next six months, they are considered not ready to self-initiate immediate changes [18]. Research has shown in a national population based sample that a stage-matched stress management intervention was effective in achieving rapid progression through the stages of effective stress management strategy adoption [18]. Given that the contemplation group for stress management in this study had high impairment costs, savings could be achieved by assisting these employees to manage or remove stress that is impacting on their work productivity. Future research should be conducted to identify if implementation of a stage of change matched stress management intervention could achieve similar results in a mining workforce as achieved in the population based sample.

Consistent with previous research that found females were more likely to exhibit help-seeking behaviours and access professional stress management or mental health support services [21,22,23], the current study found that females were significantly more likely to report wanting assistance with stress management. Although males and females had similar average impairment costs, the higher proportion of males in the mining workforce and the lower likelihood of males to want assistance suggests that practitioners need to ensure that stress management promotion is appropriate for engaging males. Male employees appear to be unlikely to initiate help seeking behaviours despite experiencing stress at work. Given that this study found that higher frequency of experiencing stress at work was associated with higher impairment costs, it was encouraging to identify that higher stress frequency was also associated with greater desire for assistance with stress management. This suggests that workplace provided stress management assistance will likely appeal to high productivity impairment cost employees that were frequently experiencing stress at work. 

Similarly, stage of change for adoption of effective stress management behaviours analyses revealed that employees in the contemplation stage had the both the highest cost impairment and highest likelihood to report wanting assistance. By contrast, employees in the pre-contemplation stage who believed their stress management habits did not need changing, were found to have both the lowest cost impairment and lowest likelihood to report wanting assistance. This again indicates that workplace provided stress management assistance will likely appeal to high productivity impairment cost employees that were intending to improve their stress management in the next six months.

### 4.1. Limitations and Future Resarch

Limitations include the recruitment process, use of self-report data, and generalizability of results. Specifically, the recruitment of participants was restricted due to the operational demands to those whom attended the work site on the day and the researchers were unable to record the number of employees who were present but declined to participate. Therefore, it was not possible to accurately evaluate the extent to which the sample reflected the wider workforce of approximately 8000 employees or response rate. Furthermore, it is not possible based on this case study to determine the extent to which the results outlined herein are reflective of employees within the broader mining industry both in Australia and globally. Additional studies are needed to longitudinally examine if demographic and work characteristics and employees’ readiness to change is associated with actual participation in workplace stress management programs. The voluntary recruitment of participants could also have exposed this study to a selection bias, with research participants potentially being more likely than the average employee to engage in healthy lifestyle behaviours.

### 4.2. Practical Implications

From a research perspective, future studies should explore whether permanent day shift and local employment are significantly associated with higher productivity impairment costs after controlling for the potential contribution of job role related responsibilities, job demands, and salaries. However, from a practical point of view, regardless of whether role or work arrangement is directly related to productivity cost impairment, based on the current findings stress management strategies should be available to all employees with a particular focus on engaging employee groups with high impairment costs. Employee groups associated with average annual productivity impairment costs in excess of $50,000 per employee included: permanent day shift employees; employees who experienced stress at work most of the time; employees who experienced stress at work all of the time; and employees who were contemplating better managing their stress in the next 6 months. To guide effective stress management in the Australian mining industry, future research should be conducted to identify whether implementation of a stage-matched stress management intervention achieves similar results in a mining workforce as achieved in a population-based sample [18].

## 5. Conclusions

To effectively design and tailor stress management strategies for a mining workforce that will deliver a high return on investment, practitioners must identify high cost employee groups and those receptive of participation in a workplace health promotion program. This study makes a novel contribution to the workplace health literature by identifying characteristics in a mining workforce associated with: (a) high stress related productivity impairment costs; and (b) characteristics of stressed employees who desire assistance with stress management in an Australian mining company.

Overall, it is likely that the observed high productivity impairment costs associated with roster and residential status (i.e., permanent day workers and local residents) is reflective of employee job roles within these groups which may include persons who reside locally and are employed in supervisory or management roles. Therefore, a targeted workplace stress management program aimed at employees in such roles may result in the greatest return on investment.

Stage of change for stress management reflects an individuals’ readiness to change and desire for assistance with stress management. According to the Stages of Change Model, individuals in the precontemplation and contemplation stages are not attempting to manage their stress. Only 13.2% of employees in the precontemplation stage and 52.1% of employees in the contemplation stage reported wanting assistance with stress management. Therefore, workplace health promotion programs targeting stress management must, in the first instance, convince employees of the value and benefit of participation in order to ensure high levels of enrolment that would result in the greatest benefit for employees and return on investment for the organisation.

Overall, these findings suggest that, within the organisation presented in this study, workplace provided stress management assistance will likely appeal to over a third of the high the productivity impairment cost employees. Furthermore, strategically targeted health promotion will be required to engage the remainder of the stressed employees with high productivity impairment costs and low desire for stress management assistance.

## Figures and Tables

**Table 1:** Participant employment characteristics.

Characteristic		*n*	%
Roster (*n* = 892)	Permanent day Shift	456	50.9
	Rotating/alternating shift	440	49.1
Employment Status (*n* = 888)	Permanent contract	777	87.5
	Contractor	111	12.5
Living arrangement (*n* = 889)	Resident	693	78.0
	FIFO/DIDO	196	22.0

Notes. *n* = 897; FIFO/DIDO = Fly-in, Fly-out/Drive-in, Drive-out.

**Table 2:** Group differences on work impairment percentage and annual productivity cost per person.

		Work Impairment (%)		Productivity Cost ($)		*t*-Value	*p*-Value
	*n*	M	SD	M	SD		
Gender							
Male	265	33.1	23.0	$44,549.93	$31,023.54	68	0.499
Female	110	34.8	22.1	$46,904.83	$29,822.75		
Roster							
Permanent day shift	206	37.6	23.5	$50,915.48	$31,501.24	3.86	<0.001
Rotating shift	169	28.7	20.9	$38,624.79	$28,134.91		
Contract							
Permanent employee	324	34.6	22.7	$46,700.16	$30,578.24	2.65	0.008
Contractor	48	25.4	21.1	$34,285.68	$28,446.86		
Residency status							
Resident	282	35.4	23.1	$47,776.63	$31,173.21	2.82	0.005
FIFO/DIDO	88	27.7	20.6	$37,295.35	$27,811.39		

Notes. *n* = 375; FIFO/DIDO = Fly-in, Fly-out/Drive-in, Drive-out; Differences in group sample size (*n*) is due to missing data on employee characteristics.

**Table 3:** Group differences based on work impairment percentage and annual productivity cost.

		Work Impairment (%)		Productivity Cost ($)		*F*-Ratio	*p*-Value
	*n*	M	SD	M	SD		
Age (years)							
Under 18 to 24	41	30.3	21.2	$40,895.44	$28,529.37	0.74	0.565
25–34	131	35.5	22.8	$47,915.20	$30,745.12		
35–44	75	34.0	22.0	$45,862.50	$29,635.07		
45–54	64	33.0	22.0	$44,478.72	$29,591.15		
55 and over	27	29.2	25.1	$39,336.96	$33,828.53		
Stress level							
Some of the time	212	27.3	18.4	$36,830.36	$24,793.38	20.61	<0.001
A good bit of the time	100	36.2	22.5	$48,764.85	$30,300.98		
Most of the time	44	48.0	22.7	$64,757.59	$30,594.89		
All of the time	19	55.9	35.2	$75,337.16	$47,379.12		

Notes. *n* = 375; FIFO/DIDO = Fly-in, Fly-out/Drive-in, Drive-out; Differences in group sample size (*n*) is due to missing data on employee characteristics.

**Table 4:** Work impairment percentage and annual productivity cost per person based on stage of change.

Stage of Change		Work Impairment (%)		Productivity Cost ($)	
	*n*	M	SD	M	SD
*Pre-contemplation—*As far as I’m concerned my stress management habits do not need changing	106	24.0	21.4	$32,284.58	$28,803.25
*Contemplation—*I’m seriously intending to better manage my stress in the next 6 months	71	39.9	19.9	$53,761.73	$26,829.95
*Preparation*—I have definite plans to better manage my stress in the next month	20	34.7	19.1	$46,702.66	$25,708.73
*Action—*I am doing something to better manage my stress	85	33.3	19.4	$44,922.71	$26,106.93
*Maintenance—*I took action more than 6 months ago to better manage my stress and I’m working hard to maintain it	30	33.9	24.8	$45,691.78	$33,484.98

Notes. *n* = 375.

**Table 5:** Logistic regression predicting the likelihood of desiring assistance with stress management.

		Do Not Want Assistance (*n* = 270; 72%)	Want Assistance (*n* = 105; 28%)					
	*n*	% or M (SD)	% or M (SD)	OR	95% C.I. (OR)			*p*-Value
Step 1—Employee and work characteristics								
Gender								
Male †	265	76.6%	23.4%	-				
Female	110	60.9%	39.1%	2.27	1.27	-	4.08	0.006
Age	338	36.66 (11.74)	36.44 (9.99)	1.01	0.98	-	1.03	0.697
Roster Type								
Permanent Day Shift †	206	66.5%	33.5%	-				
Rotating/Alternating	169	78.7%	21.3%	0.81	0.43	-	1.54	0.515
Contract Type								
Employee †	324	69.4%	30.6%	-				
Contractor	48	87.5%	12.5%	0.41	0.13	-	1.35	0.139
Residency Status								
Resident †	282	69.9%	30.1%	-				
FIFO/DIDO	88	78.4%	21.6%	1.10	0.50	-	2.30	0.809
Stress Levels								
Some of the time	212	83.5%	16.5%					
A good bit of the time	100	60.0%	40.0%	3.18	1.66	-	6.08	<0.001
Most of the time	44	47.7%	52.3%	6.47	2.78	-	15.03	<0.001
All of the time	19	63.2%	36.8%	4.45	1.21	-	16.37	0.025
Step 2—Stage of Change								
Pre-contemplation †	106	86.8%	13.2%	-				
Contemplation	71	47.9%	52.1%	11.05	4.26	-	28.70	<0.001
Preparation	20	60.0%	40.0%	7.13	1.97	-	25.87	0.003
Action	85	77.6%	22.4%	2.85	1.07	-	7.62	0.037
Maintenance	30	66.7%	33.3%	5.22	1.60	-	16.99	0.006

Notes. *n* = 375; Differences in group sample size (*n*) is due to missing data on employee characteristics; logistic regression sample included in analysis *N* = 275 due to missing values; FIFO/DIDO = fly-in, fly-out/DRIVE-in, drive-out; OR = odds ratio; CI = confidence intervals; † Reference.
